# Editorial: Women in aging neuroscience 2021

**DOI:** 10.3389/fnagi.2023.1140899

**Published:** 2023-01-26

**Authors:** Silvia Fossati

**Affiliations:** Alzheimer's Center at Temple, Department of Neural Sciences, Temple University, Philadelphia, PA, United States

**Keywords:** female neuroscientists, sex differences, biomarkers, psychosocial evaluation, therapeutic strategies, neurodegenerative disorders

## Introduction

While the number of women in neuroscience is gradually increasing, reaching over 50%, the proportion of women in aging neuroscience at higher career levels, such as Professors and Chairs, is still severely discrepant compared with their male counterpart. This is known as the “leaky pipeline,” likely due to a combination of personal factors as well as a recognized gender inequality in the possibility to obtain grants and publish manuscripts in high impact journals (https://doi.org/10.1016/B978-0-12-819641-0.00007-4; https://www.frontiersin.org/articles/10.3389/fpsyg.2019.01297/full). While solving these well-recognized issues will require longer time frames, this Research Topic aims to highlight the wonderful and impactful work of women-neuroscientists.

Our contributors are female authors that work on aging neuroscience as well as neurodegenerative disorders. Even if still outnumbered by their male counterparts, these women are among the current and future leaders in the neuroscience and neurodegeneration fields. This important collection of papers clearly indicates that these impactful scientists, together with other established female colleagues, will represent influential role models to lead the path to a bright future for the next generation of women in aging neuroscience.

This diverse group of manuscripts highlights the high level of research lead by women neuroscientists. Interestingly, in some cases, this Research Topic also unveils differences in the incidence and causes of neurodegenerative disorders, such as Alzheimer's (AD) and Parkinson's disease (PD), in women compared to males, tackling the subject *of* “women in aging neuroscience” from two complementary sides.

## Effect of hormones and menopause on AD and PD risk

One important aspect of sex differences in neurodegenerative diseases is due to the impact of steroid hormones and menopause on the risk to develop these age-associated disorders.

Two manuscripts on this subject were led respectively by Dr. Lisa Mosconi, and by Dr. Roberta Marongiu. Jett et al. elegantly summarize how 17β-estradiol, an ovarian hormone with multiple neuroactive properties, which has been called “the master regulator of the female brain,” is involved in the neurobiology of aging, AD and cognitive impairment. The authors remind us that estradiol levels drop at menopause, in association with complaints of poor sleep, loss of thermoregulation, and impaired cognitive abilities. Importantly, as shown by Dr. Mosconi and others, this was also linked to a disproportionate increase in AD-related imaging biomarkers in the brain of peri- and post-menopausal women compared to age-matched men. Especially, women at risk for AD, exhibited preclinical AD endophenotypes already during perimenopause. The fact that women constitute about 2/3 of AD patients (not only due to their longer life expectancy; https://www.alz.org/media/documents/alzheimers-facts-and-figures.pdf), highlights the importance of better understanding menopause-AD relationships. However, the debate remains open on whether menopausal hormone therapy has neuroprotective value.

In the second manuscript, Unda et al. tackle a similar subject in relation to PD. Interestingly, the authors acknowledge that women have a lower risk for PD compared to men, differently from AD, which raises the possibility that menopause and ovarian hormones may have very different mechanistic effects in brain areas more affected in AD or PD. The authors clarify that, due to the heterogeneity in study designs, the role for age at menopause, type of menopause and hormone replacement therapy on PD is still unclear. This study, while collecting the newest information on the subject, also highlights gaps in the literature and provides indications for the best ways to answer some of these open questions.

## Development of Medication Adherence Scales for PD

Since suboptimal medication adherence in neurodegenerative diseases remains a problem, Tosin et al. outlined an objective design of the development and validation of the PD Medication Adherence Scale. This report demonstrates the feasibility of such an instrument for medication adherence process in people with PD, allowing the proper implementation of these techniques.

## Stress, loneliness, and isolation factors in aging and dementia

Other manuscripts tackle the important subjects of psychosocial evaluations, stress, loneliness and isolation in elderly people with dementia. In the first of these papers, Wuttke-Linnemann et al. interviewed patients and caregivers in a day clinic context. This work is timely, as measure of success of AD/dementia therapies, especially for the psychosocial response, is often based on caregivers opinions. The authors highlight the possibility that physiological stress markers, such as hair cortisol, could help in complementing the evaluation of treatment effects. Not all participants were willing to collect physiological stress markers. However, hair samples were easier to obtain than saliva. Because of the inability or unwillingness for people with dementia to correctly describe their symptoms, collecting these biomarkers may be an important strategy to accurately measure stress values in research or clinical trials subjects.

In the opinion article, Morese and Palermo highlight how the impairment of cognitive function may have an impact on loneliness for older people, through changes in interaction with family and friends, or the perception of relationship satisfaction. Social isolation during life may also contribute, through induction of depression and other described biological mechanisms, such as inflammation, to an increased risk of AD and dementias. Notably, loneliness is a modifiable factor, which can be addressed during life to prevent the development of cognitive impairment and AD-related dementias.

In apparent contrast, the manuscript of Bouter and Bouter suggests that in cognitively impaired and cognitively normal participants from the Alzheimer's Disease Neuroimaging Initiative (ADNI) study, serotonin reuptake inhibitors (SSRI)-treatment, associated with depression improvement, did not show beneficial effects on amyloid load nor cognition. This may indicate that depression or social isolation must be treated earlier in life to prevent cognitive dysfunction, rather than as a therapy. Overall, controlled randomized prospective studies on the effect of SSRIs on AD-pathologies are necessary to overcome limitations of previous studies.

## Relationship of sympathoexcitatory responses with white matter hyperintensities

In another valuable contribution of Pearson et al., the association between cardiovascular and cerebrovascular responses in hand grip exercises, post-exercise ischemia, and white matter hyperintensities (WMH) were assessed. This study suggests that individuals who show smaller increases in responses to sympathoexcitatory stress have greater WMH burden, strongly associating weaker peripheral cardiovascular responses to cerebrovascular dysfunction and WMH.

## Changes in comprehension and CSF metals in neurodegeneration

This Research Topic spanned over multiple types of dementia. In Falque et al., comprehension impairment was found in mild dementia with Lewy bodies (DLB) subjects. There was also a correlation between striatal gray matter volumes and DLB patients' ability to organize information. This work points to the need of future research on the association of the striatum and striato-frontal processes with narrative comprehension in dementias.

Finally, the study, Chen et al. aimed to examine potential associations between cerebral spinal fluid (CSF) metals and amyotrophic lateral sclerosis (ALS) risk. The study found that Cu levels were lower in the ALS and spinal-onset groups, while Ni levels were higher in the spinal-onset group compared to the control and bulbar-onset groups, highlighting the differential association of CSF metals with neurodegeneration.

## Author contributions

The author confirms being the sole contributor of this work and has approved it for publication.

